# Chronic Gonorrhea in the Female

**Published:** 1911-01

**Authors:** 


### Chronic Gonorrhea in the Female.

[*The Hospital*, November 5, 1910.]

THAT chronic gonorrhea is far less uncommon in the female than one might suppose from the accounts given in the textbooks is being made increasingly obvious by modern publications upon the subject; and seeing that even a slight gleet on the part of the husband may lead to recurrent infection of the wife, and thus to chronic gonorrhea, without there having been any definite acute gonorrhea, it is not surprising that the chronic form of the disease is not uncommon in females.

### A NOTE ON TREATMENT.

In regard to its treatment, if there are active symptoms the patient should be kept absolutely in bed for the time being. Coitus must be forbidden, and if the husband shows any signs of the disease he must be treated for it actively either by the use of intraurethral injections or by means of vaccines, or by both. The diagnosis having been confirmed by a thorough examination, and, if possible, by the detection of the gonococcus in films from any of the infected parts, gonococcal vaccine treatment should be adopted in the woman also; but rather than rely upon vaccines only the action of the latter should be assisted in every possible way besides. The diet should be bland but generous, bland in kind but generous in amount, including plenty of proteid, especially in the form of milk. It is inadvisable to use the curet except in special conditions, and medical treatment will often suffice without operative measures.

### MECHANICAL AND ANTISEPTIC CLEANSING.

Dr. Lochrane advises that the vulva should first be cleaned thoroughly by washing with soap and water and then with a solution of biniodide or perchloride of mercury, a vaginal douche of biniodide of mercury solution, 1 in 4,000, given daily for four days. Bartholin's glands are generally involved, and their ducts should be swabbed each day with some antiseptic such as a 20 per cent. solution of argyrol on sterilised cottonwool and a probe. In resistant cases it may be necessary to lay open the ducts of these glands and pack them with gauze soaked in a 5 per cent. solution of argyrol. The cervix uteri must be exposed, the cervical canal swabbed free from discharge, any Nabothian follicles present should be laid open, and any erosion should be treated with some astringent antiseptic such as a 20 per cent. solution of picric acid in alcohol. The mucous membrane of the cervical canal as far as the internal os is then thoroughly swabbed with a solution of 20 per cent. of argyrol, which has the advantage over other preparations of being relatively soluble, highly bactericidal, and non-irritating.

DOUCHING AND CURETING.

Lochrane advises that any excess of solution should be wiped away and a small pencil-shaped piece of fresh bakers' yeast inserted into the mouth of the cervical canal, a few grains of ordinary cane sugar being inserted after it. The yeast grows upon the sugar and has a remarkable destructive effect upon the gonococcus and other pyogenic cocci. A large piece of fresh yeast with a little cane sugar may be left against the cervix. This treatment may be repeated every day for seven days and then stopped, the discharge being re-examined for gonococci. If the disease has not reached the endometrium the condition may be entirely cured by the above measures, and in any case it is important not to dilate the internal os or to pass anything upward beyond it lest the disease should be spread to the interior of the uterus. If, however, the interior of the uterus has already become involved before the patient comes under treatment, it may be necessary to dilate the cervical canal and swab out the interior of the uterus likewise with argyrol solution, beginning with half the strength used in the cervical canal, but gradually increasing until even 20 per cent. may be reached, provided no increase is produced in the endometritis. Curetting of the interior of the uterus and cervical canal should only be resorted to if prolonged rest, diet and treatment as above by argyrol, yeast, and sugar do not result in cessation of the profuse discharge and heavy loss at the monthly periods. There is always some risk of extending the disease if the measures adopted are too active. If relief has been partial only, it is better to wait for a month or more, seeing the patient at intervals, because it not infrequently happens that when the active measures have brought some relief the cure continues for some time afterwards even though no further active measures are taken. At most the vagina should be douched with plain hot water in the morning, and if there is any chronic discharge, as is often the case, this may be minimised by the use of vaginal suppositories containing 10 grains each of tannin and boric acid. The urethra in the female is very seldom attacked, or at least not to the extent that it is in the male, and when it is involved at all the inflammation affects chiefly Skene's glands and their ducts. The latter open immediately inside the urethral meatus in the lower part of its lateral aspect, and their inflammation is indicated by a pouting of the mucous membrane at the meatus, the inflamed duct-openings being visible as two small red dots on the pouting membrane. Argyrol solution in a strength of about 10 per cent. may be used for disinfecting these either by application to their surfaces or by injection by means of a suitable nossle syringe, or even, if necessary, by opening up the ducts.

### EXTENSION PROCESSES.

It is remarkable how immune to the gonococcus the walls of the vagina itself seem to be, though there is very apt to be extension of the disease to the Fallopian tubes, the broad ligament, and to Douglas's pouch of the peritoneum, with resultant pain in the lower part of the back and dysmenorrhea. If there is actual pus in the Fallopian tubes, it is improbable that perfect relief will be effected by any other measures than removal of the affected parts; nevertheless there is the danger of producing peritonitis by the necessary manipulations, and this same danger applies also to cases in which pregnancy occurs in conjunction with gonococcal inflammation of the pelvis. Indeed it is not improbable that at least a few of the cases of general peritonitis that result from labor are due less to the introduction of microörganisms by the hands of the midwife or physician than to the letting loose of organisms that have been pent up in local inflammatory and possibly gonococcal lesions in the neighborhood of the uterus.

### RESULTS OF MEDICAL TREATMENT.

It is astounding what good results have been obtained in Germany by the medical treatment alone. Rest for even as long as six months, with careful dieting, the application of argyrol and glycerin, and the alternate application of heat and cold to the parts by the use of hot air, or hot douches on the one hand and ice-bags and wet compresses to the hypogastrium and perineum on the other, have led to the destruction of pelvic exudates of a chronic gonorrheal nature. Nevertheless, some of these lines of treatment are in themselves distasteful to the patient, and in this country, at any rate, if rest in bed and medical treatment do not result in a cure of the pelvic condition fairly readily it is probable that in the majority of cases some operative measures will be resorted to, especially if there is pyrexia or other evidence of a continuation of what may be a dangerous inflammation.

